# Mechanical Resistance of the Largest Denticle on the Movable Claw of the Mud Crab

**DOI:** 10.3390/biomimetics8080602

**Published:** 2023-12-13

**Authors:** Tadanobu Inoue, Yuka Hara, Koji Nakazato

**Affiliations:** National Institute for Materials Science, 1-2-1, Sengen, Tsukuba 305-0047, Japan; hara.yuka@nims.go.jp (Y.H.); nakazato.koji@nims.go.jp (K.N.)

**Keywords:** abrasion resistance, nanoindentation, elemental composition, tissue structure, biomineralization

## Abstract

Decapod crustaceans have tooth-like white denticles that are present only on the pinching side of the claws. In the mud crab, *Scylla serrata*, a huge denticle exists on the movable finger of the dominant claw. This is mainly used to crush the shells of the crab’s staple food. The local mechanical properties, hardness (*H_IT_*) and elastic modulus (*E_r_*), of the peak and valley areas of the largest denticle were examined via a nanoindentation test. The microstructure and elemental composition were characterized using a scanning electron microscope and energy-dispersive X-ray spectroscopy. The striation patterns originating from a twisted plywood structure parallel to the surface were visible over the entire denticle. Most of the largest denticle was occupied by a hard area without phosphorus, and there was a soft layer corresponding to the endocuticle with phosphorus in the innermost part. The *H_IT_* of the denticle valley was about 40% lower than that of the denticle peak, and the thickness of the soft endocuticle of the denticle valley was five times thicker than that of the denticle peak. The *H_IT_*–*E_r_* map showed that the abrasion resistance of the denticle surface was vastly superior and was in the top class among organisms. The claw denticles were designed with the necessary characteristics in the necessary places, as related to the ecology of the mud crab.

## 1. Introduction

The advent of new material-processing technologies such as additive manufacturing has made it possible to fabricate materials with complex biological structures found in living organisms [1,2,3,4,5,6,7]. Therefore, elucidation of the microstructure, structure, and characteristics of various organisms is underway with the aim of improving the characteristics and functions of fabricated materials [8,9,10,11,12,13,14,15,16,17,18,19].

Based on the maximum force (biting, pinching, swimming, jumping, running, and so on) exerted by animals [20,21], the maximum force per unit body weight (*F*/*BW*) ranges from 0.5 *BW*^−1/3^ to 20 *BW*^−1/3^; the maximum force, *F*, shows a negative correlation with *BW*. Most animals, including humans, have a force several times their body weight, but the (*F*/*BW*) of the crustaceans greatly exceeds that of other animals. Hence, the tissue and mechanical properties of the claw exoskeleton of crustaceans have been studied often [1,9,12,22,23,24,25,26,27]. The claws have tooth-like white denticles that are the first point of contact when crustaceans pinch their prey. Curiously, few studies have examined the tissue and mechanical properties of the denticles as compared with the exoskeleton of the carapace and claw. Waugh et al. [28] examined the denticle microstructures of *Scylla serrata* and *Callinectes sapidus* and compared them to the cuticle in the remainder of the claw. Denticles were differentiated from the surrounding cuticle and were composed primarily of a modified endocuticle, with little or no epi- or exocuticle present in this region in crabs. In addition, Waugh et al. [28] mentioned that the increased mineralization of claw tips and denticles may make them even more durable than the chelae. Rosen et al. [29] examined the denticles of two anomuran crabs and three brachyuran crabs; they were the first to note that the hardness of the denticles was higher than that of the endocuticle, that there was a decrease in the phosphorous (P) content, and that a tissue change from the endocuticle to the denticle was visible. In our previous paper [27], we showed that the mechanical properties, elemental composition, and tissue structure of the claw of the coconut crab, *Birgus latro*, changed significantly from the denticle to the endocuticle. The endocuticle had a twisted plywood structure parallel to the surface. On the other hand, the denticle was composed of a columnar structure vertical to the surface. This feature was also observed in the denticles of anomuran crabs and brachyuran crabs [29].

**Figure 1:**
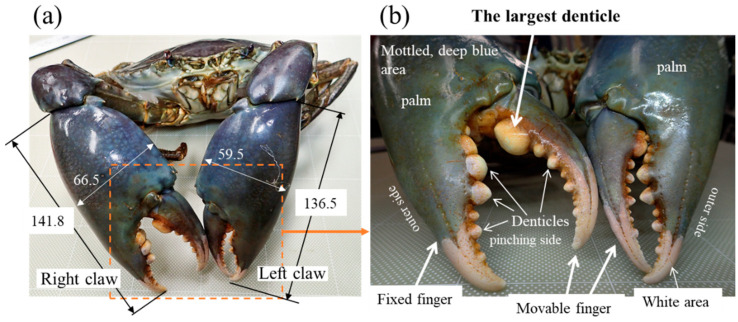
Photographs of (**a**) the mud crab used in the present study and (**b**) enlarged claws.

The mud crab, *Scylla serrata*, is the strongest crustacean in brackish water and is more ferocious than the coconut crab, one of the largest terrestrial crustaceans. In addition, the mud crab can grow up to 300 mm in carapace width and 2.5 kg in weight [30,31,32] and has extremely large/robust claws. The denticles of the mud crab are arranged in a line on the claw’s fingers [28,33], and the denticles get smaller from the palm to the fingertip (Figure 1). In particular, the largest denticle exists at the base of the movable finger of the larger claw (dominant claw). The mud crab places a staple food (shellfish) on two large denticles on the base of the fixed finger, crushes the shells with the largest denticle, and can consume the contents. For the mud crab, the loss of the largest denticle is directly linked to the inability to consume the staple food, so it is considered that the abrasion resistance here is the highest in the entire body. Biomineralized complex tissues and structures provide valuable data for design concepts for man-made materials. Understanding the relationship between microstructure, elemental composition, and mechanical properties of the exoskeleton and denticle of the mud crab and comparing the results with those of various organisms would lead to the development of bulk materials with strong/tough and excellent abrasion resistance in the future.

In this paper, the local mechanical properties of the largest denticles on the movable finger of the large claw of the mud crab were investigated via a nanoindentation test. The elemental composition and microstructure were characterized using a scanning electron microscope (SEM) and energy-dispersive X-ray spectroscopy (EDS), and their variations related to the mechanical properties were discussed. The results were compared with the abrasion resistance of the endocuticle, the small and large denticles on the fixed finger, and the fingertip of the claw, as well as with other organisms.

## 2. Materials and Methods

### 2.1. Sample Preparation

**Figure 2:**
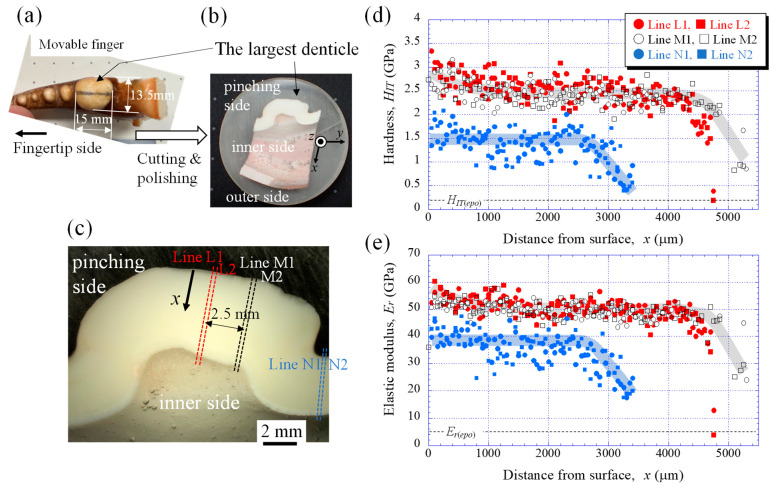
(**a**) Movable finger of the mud crab, (**b**) the claw cross section after polishing, and (**c**) optical micrographs of a cross-section of the largest denticle. Distributions of (**d**) hardness, *H_IT_*, and (**e**) elastic modulus, *Er*, with distance from the surface, *x*, on Lines L1, L2, M1, M2, N1, and N2. Here, *H_IT_*_(*epo*)_ and *E_r_*_(*epo*)_ denote the hardness and elastic modulus of the cold epoxy resin, respectively.

A large living male mud crab was obtained from a local market in Okinawa, Japan (Figure 1a). The body weight of the crab was 1265 g, and the carapace width and length were 167.3 mm and 113.4 mm, respectively [33]. Since the sample animal was a wild crab, the exact number of days after molting was unknown, but it could be estimated based on the color of the shell (from whitish to deep blue). Under the guidance of a mud crab expert [34], the sample was deemed to have a sufficiently hard exoskeleton after molting and was an adult crab estimated to be over 2 years old. Before freezing the crab, general anesthesia was applied by dipping the crab into cold ice water (approximately 0–4 °C) to minimize its suffering. To prevent natural decay processes, the crab was stored frozen at −18 °C and transported to Tsukuba. The large right claw shown in Figure 1 was thawed under running water, the movable finger was broken by applying a force opposite to the range of motion (Figure 2a), and a sample containing the largest denticle was cut from the movable finger using a handsaw. First, the sample was hand-polished to expose the cross sections near the middle of the large denticle. The mounting cup in which the sample was set was then filled with epoxy, EpoFix Resin (Struers, Tokyo, Japan), and left to cure at room temperature for 12 h. Many voids were observed in the epoxy resin, so the sample was kept under vacuum for 600 s soon after the epoxy was added. Subsequently, the sample was ground with SiC papers, polished with 9, 3, and 1 μm diamond suspensions, and finally polished with a 0.05 μm alumina suspension (Figure 2b). A cross-sectional image of the largest denticle taken using a digital microscope, VHX-900 (Keyence, Osaka, Japan), is shown in Figure 2c.

### 2.2. Nanoindentation Tests

The mechanical properties were examined via nanoindentation testing using an ENT-NEXUS (ELIONIX, Tokyo, Japan) with a Berkovich diamond indenter. The test was performed at ambient temperature after polishing. The loading curve consisted of a loading/unloading rate of 1 mN s^−1^ with a holding time at the maximum force of 5 mN for 5 s. The tests were conducted at intervals of 50 μm from the outer surface to the inner surface of the cross-section along Lines L1, L2, M1, and M2 (denticle area) and Lines N1 and N2 (valley area), as shown in Figure 2c. Here, each letter, i.e., L, M, and N, denotes a grouping of two parallel lines that are 100 μm apart. As a result, the nanoindentation tests were performed 95 times each for Lines L1 and L2, 106 times each for Lines M1 and M2, and 68 times each for Lines N1 and N2. The hardness (*H_IT_*) and reduced elastic modulus (*E_r_*) were analyzed from the unloading curve using the Oliver–Pharr method [35] employed in biological studies [9,11,22,26,36].

### 2.3. Microstructure Observation

After nanoindentation tests, the entire cross-section of the sample was coated with approximately 2 nm of osmium using a Neo Osmium Coater (Meiwafosis, Tokyo, Japan) to characterize the microstructure and elemental compositions. A focused ion beam (FIB)–SEM dual-beam instrument, Scios2 (Thermo Fisher Scientific, Waltham, MA, USA), at an accelerating voltage of 2 kV with a secondary electron detector in a chamber or an annular in-lens backscattered electron detector was used for the microstructure characterization. An EDS device attached to this FIB–SEM instrument was applied for the compositional analysis. High detection efficiency and low statistical error in the quantitative analysis were ensured by using a large silicon-drift detector, Ultim Max 170 (Oxford Instruments, Abingdon, Oxfordshire, UK). The EDS analysis was performed at an accelerating voltage of 15 kV. The EDS measurements were taken near the inner surface of Lines L1 and N1 and near the denticle surface. The EDS results showed carbon (C), oxygen (O), calcium (Ca), magnesium (Mg), P, sodium (Na), chlorine (Cl), aluminum (Al), and sulfur (S). Because the sample was polished with a 0.05 μm alumina suspension, Al was excluded from the EDS quantitative analysis.

## 3. Results

### 3.1. Mechanical Properties

Figure 2d,e show the variations in *H_IT_* and *E_r_* with distance from the outer surface, *x*, in the denticle and valley areas. On Lines L1, L2, M1, and M2 of the denticle area, the *H_IT_* gradually decreased with *x*, became constant (2.5 GPa) at *x* = 600 µm, and then decreased again from *x* = 4000 mm. On the other hand, on Lines N1 and N2 of the valley area, the *H_IT_* became constant (1.6 GPa) until *x* = 2600 µm and then decreased with *x*. Note that the data points in the valley area are relatively sparse as compared to those in the denticle area. The *E_r_* also showed the same tendency throughout the cuticle thickness. Although no clear difference in the tissue was visible at the optical microscope (OM) level, as shown in Figure 2c, there was a difference in the mechanical properties between the denticle and valley areas on the pinching side. In short, the largest denticle has the mechanical properties of (*H_IT_*, *E_r_*) = (2.5 GPa, 50 GPa), the outermost surface is higher, and the area adjacent to the cells is lower. The valley area is (*H_IT_*, *E_r_*) = (1.5 GPa, 38 GPa) and the area adjacent to the cells becomes lower.

### 3.2. Microstructure and Elemental Composition

**Figure 3:**
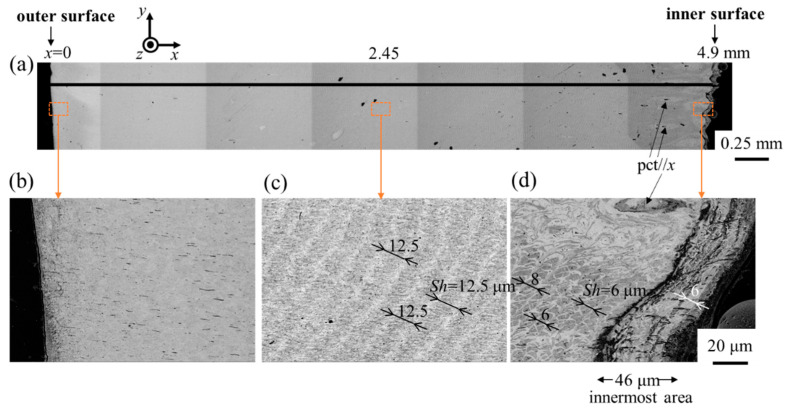
(**a**) SEM images of Line L. Enlarged views of (**b**) near the outer surface, (**c**) the middle, and (**d**) the inner surface. Here, *Sh* denotes the stacking height of a twisted plywood-pattern structure and pct denotes pore canal tubes.

Figure 3 shows SEM images of Line-pair L, shown in the finger cross section in Figure 2c, and enlarged views of the areas near the outer surface, the center of thickness, and the inner surface. The microstructure near the outer surface (Figure 3b) could not be characterized via SEM, but striation patterns originating from a twisted plywood structure [1,9,12,26,33] were observed as the observation position progressed inward (Figure 3c,d). As shown in Figure 3d, pore canal tubes (pct/*x*) were observed in the microstructure near the inner surface and the innermost layer with black streaks of about 46 μm was visible. Since this layer has striation patterns similar to those of the middle tissue, this is not the membranous layer. Although the microstructure near the outer surface is not clear, the striation patterns parallel to the surface are wavy from the center of the denticle to the inner surface, and the stacking thickness (*Sh*) of the plywood structure on the inner side is smaller than in the center.

**Figure 4:**
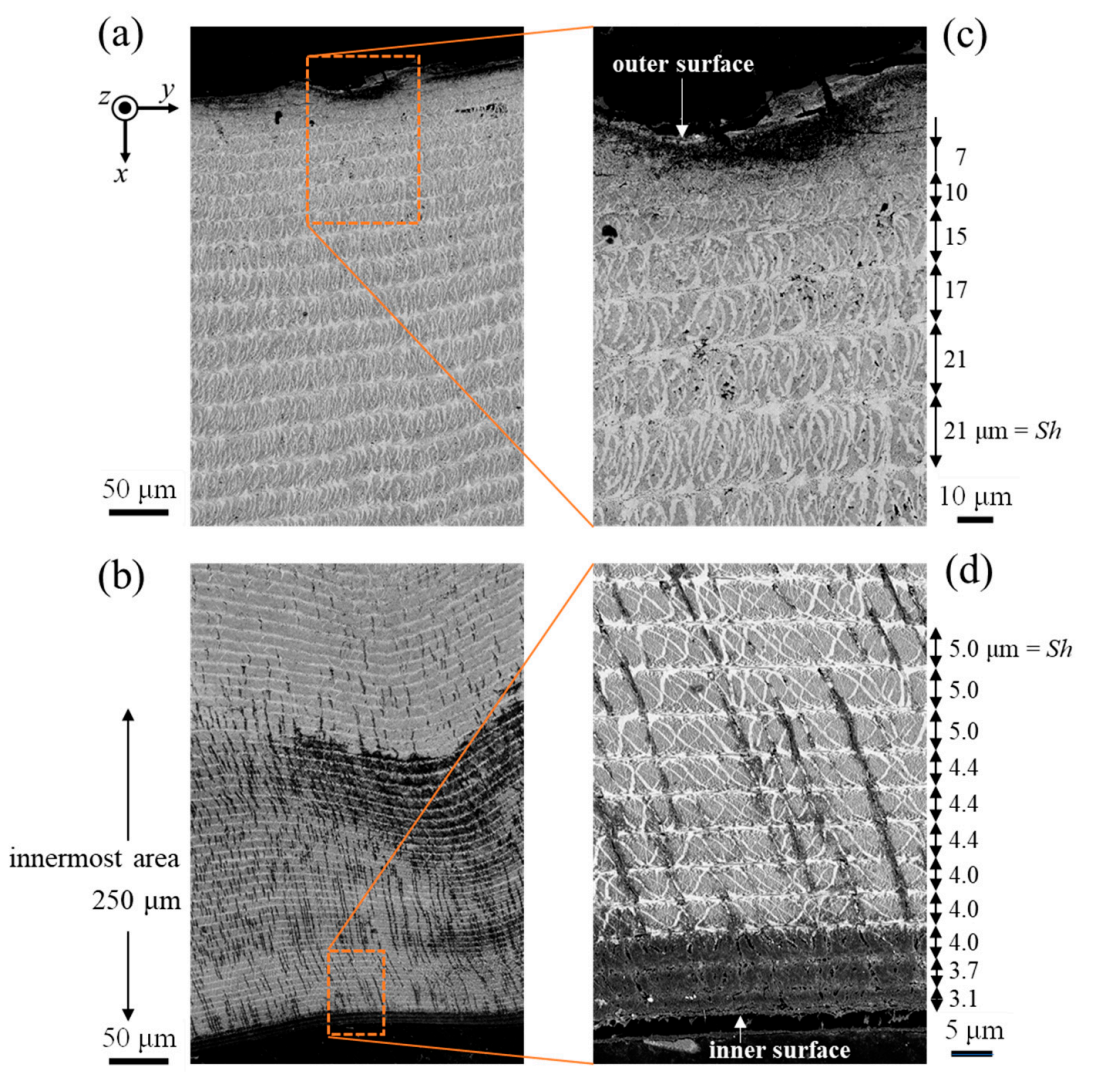
SEM images of Line N (**a**) near the outer surface and (**b**) on the inner surface. (**c**,**d**) Enlarged views of the areas surrounded by rectangles in (**a**,**b**). Here, *Sh* denotes the stacking height of a twisted plywood-pattern structure.

The SEM images of Line-pair N, corresponding to the valley area, are shown in Figure 4. Unlike the denticle area, the striation patterns in the valley area could clearly be observed throughout the cuticle thickness. The *Sh* gradually decreases as it approaches the outer and inner surfaces. The *Sh* near the inner surface is very small, at 3.1 μm. In the SEM images showing the area near the inner surface in Figure 4b,d, the area with black streaks with different levels of contrast was observed in a layer approximately 250 μm thick from the innermost surface.

**Figure 5:**
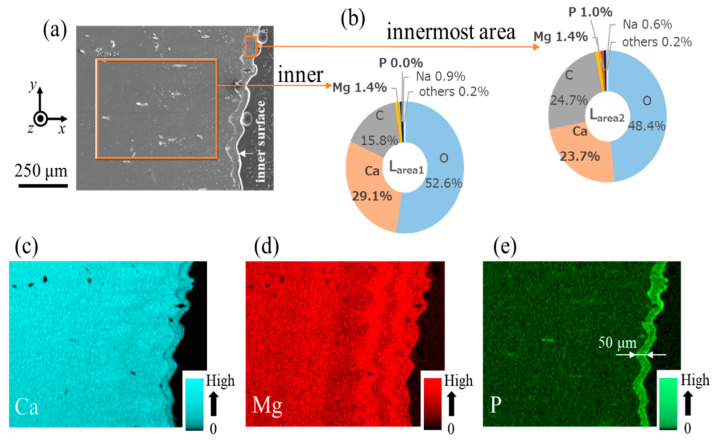
(**a**) SEM images near the inner surface of Line L1 of the denticle area. (**b**) EDS area scan results at the region surrounded by orange rectangles, where the EDS results show the average weight % of Ca, Mg, P, C, O, Na, and others. EDS maps showing the distribution of (**c**) Ca, (**d**) Mg, and (**e**) P.

**Figure 6:**
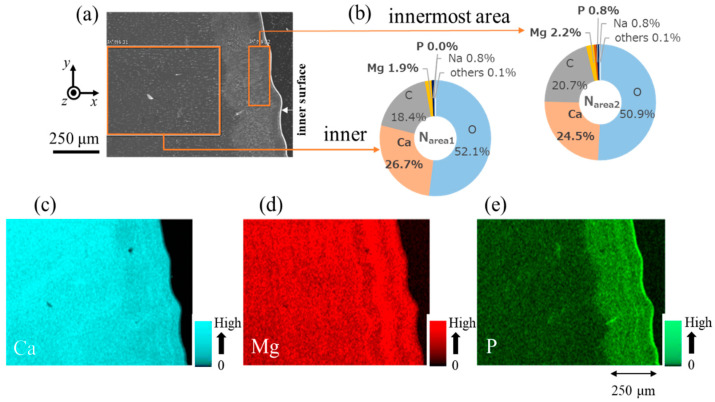
(**a**) SEM images near the inner surface of Line N1 of the valley area. (**b**) EDS area scan results at the region surrounded by an orange rectangle, where the EDS results show the average weight % of Ca, Mg, P, C, O, Na, and others. EDS maps showing the distribution of (**c**) Ca, (**d**) Mg, and (**e**) P.

Figure 5 shows the EDS results near the inner surface on Line L1. Since no significant change in composition was observed inside the denticle, only the results for the inner surface are shown. The EDS maps shown in Figure 5c–e clarified the difference in composition between the inner area and the innermost area. In particular, P was only detected in the innermost area, and the P concentration of the area (L_area2_) shown in Figure 5a was 1.0% (Figure 5b). The concentration of Ca in L_area2_ was lower than that in the inner area (L_area1_). Although not shown here, the EDS area scan results indicated that the inorganic component near the outer surface of Line L1 was 30.7% Ca–1.2% Mg–0% P. The EDS results for Line N1 are shown in Figure 6. As in the denticle area, the difference in composition between the inner area and the innermost area was confirmed in the valley area. The P concentration of the inner area (N_area1_) was zero, but it was 0.8% in the innermost area (N_area2_). The EDS map shown in Figure 6e revealed the difference in the P concentration between the inner area and the innermost area. The Ca concentration in N_area2_ was lower than that in N_area1_. The outer surface of Line N1 was 29.4% Ca–1.1% Mg–0% P. In Lines L1 and N1, the cuticle thickness where P exists corresponds to the layer thickness where many black streaks are observed on the innermost surface in Figure 3d and Figure 4b.

## 4. Discussion

### 4.1. Cuticle Change on the Pinching Side

**Figure 7:**
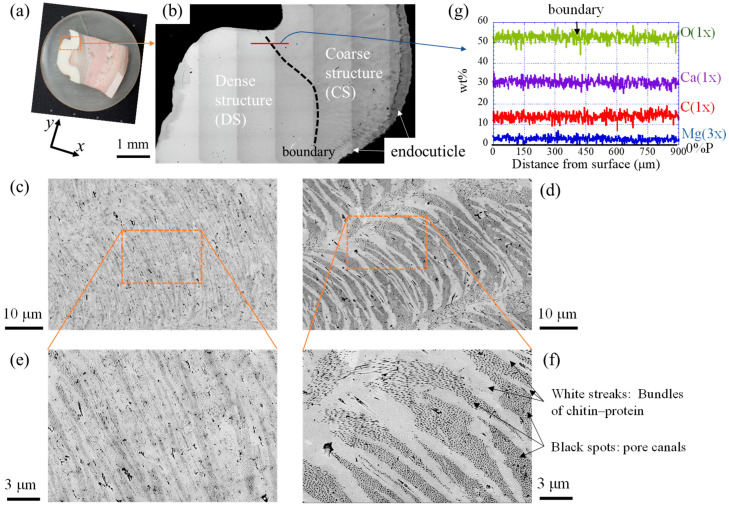
(**a**) Claw cross section after polishing and (**b**) enlarged SEM view of the area surrounded by the rectangle in (**a**). SEM micrographs of (**c**) the dense structure (DS) area and (**d**) the coarse structure (CS) area. (**e**,**f**) Enlarged views of areas surrounded by rectangles in (**c**,**d**). (**g**) Line-scanning profiles with the distance on the red line in (**b**) as measured by EDS. Here, the weight % of Mg was expanded by a factor of 3 relative to those of Ca, C, and O.

As shown in Figure 2d,e, there was a significant difference in the mechanical properties between the denticle and valley areas on the pinching side. This indicates that a boundary between those areas exists in the cuticle. Since it was difficult to determine the boundary using an OM, we examined it in detail using an SEM/EDS. The results are summarized in Figure 7. The dashed black line drawn in the SEM image of Figure 7b represents the boundary between the area where the striation patterns were observed (CS: coarse structure) and the area where they were not (DS: dense structure). In the CS shown in Figure 7d,f, the white streaks are the bundles of chitin fiber wrapped by protein and many black spots (pore canals) are visible between these streaks. Such SEM images have been observed in crustacean exoskeletons with twisted plywood-pattern structures [12,23,27,29,33,37]. On the other hand, in the DS shown in Figure 7c,e, striation patterns could be seen, but white streaks could not be observed as clearly as in the CS. Many black spots are visible in the SEM images, and they were very small compared to the black spots in the CS. Rosen et al. [29] showed that there was an increase in the packing density of chitin–protein bundles, a decrease in the width of the pore canals, and a decrease in the P concentration from the endocuticle to the denticle in the cuticle. Curiously, the EDS line profile on the red line across the boundary shown in Figure 7g shows that the components are unchanged at the boundary. Here, the average values on this line were 30.6% Ca–0.9% Mg–0% P. The P content is zero even in the valley area, as shown in Figure 6, so the CS including the valley area can be judged to be part of the denticle rather than the endocuticle. The change in mechanical properties between the DS and CS, as shown in Figure 2d,e, is a result of the tissue change from dense to coarse. Since the indentation size of the nanoindentation tests is approximately 2 to 5 μm, the variation in data for the valley area in Figure 2d,e is due to the coarse structure.

As shown in Figure 5 and Figure 6, there was an area where P was present only on the innermost surface of the denticle and the valley. The EDS results are summarized in Table 1. For comparison, the data for the fingertip, large denticle, and outer side of the fixed finger in the same claw [33,38] are also shown. The P concentrations of 0.8–1.0% in these areas agree with the P concentrations in the endocuticle of the fixed finger reported in previous papers [33,38]. Other inorganic components, 23.7–24.5% Ca and 1.4–2.2% Mg, also agree. The *H* and *E_r_* decrease in these areas, as shown in Figure 2d,e, and these magnitudes are consistent with *H* = 0.5–1.0 GPa and *E_r_* = 20–30 GPa for the endocuticle of the mud crab [33]. Hence, the area of the innermost surface with P on the pinching side is the endocuticle. In the SEM image, this area is observed as a region with black streaks, as shown in Figure 3a,d and Figure 4b,d.

Consequently, most of the largest white cuticles, like the teeth of the movable finger, are denticles without P, and there is a layer corresponding to the endocuticle with P in the innermost part. This layer is visible as an area with different levels of contrast in the SEM observation, and the endocuticle thickness of the denticle valley (250 μm) is five times thicker than that of the denticle peak (50 μm). Note that the ratio of the endocuticle thickness to the cuticle thickness in the denticle peak is only 1%, and that in the denticle valley it is 8%. These ratios are much lower than the ratio of the endocuticle thickness to the cuticle thickness on the outer side of the fixed finger of the mud crab [33] and of other crustacean exoskeletons [9,27,29]. Since a large force acts on the denticle peak, that is the first contacted part of the movable finger when the mud crab pinches prey, the cuticle at the denticle peak is very thick and hard. In the denticle valley, where the force on the pinching side needs to be relaxed, the hardness is about 40% lower than that at the denticle peak; furthermore, the ratio of the soft endocuticle increases. The claws evolved to have the necessary characteristics in the necessary places, as related to the ecology of the mud crab.

### 4.2. Hardness (H_IT_)–Elastic Modulus (E_r_) Map

**Figure 8:**
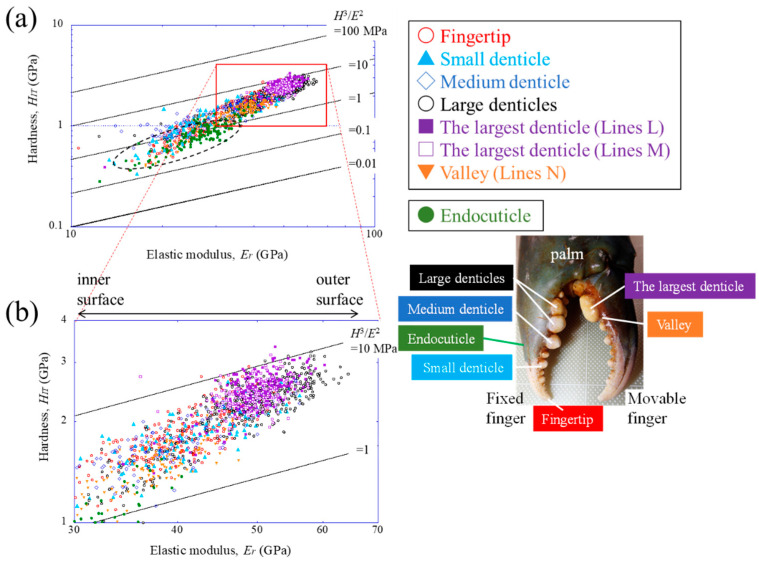
(**a**) The *H_IT_*–*E_r_* map on Lines L1, L2, M1, M2, N1, and N2, as shown in Figure 2d,e, and (**b**) an enlarged map of the area near the maximum value of the *H_IT_*–*E_r_* balance. For comparison, data for the fingertip, endocuticle, and denticles of the fixed finger were also plotted [32,37]. Here, data lying on a straight line of *H*^3^/*E*^2^ indicate materials with equivalent abrasion-resistance performances.

To understand the mechanical resistance of the largest denticle, the results were plotted on a hardness–elastic modulus related to the abrasion resistance of the materials [39]. Figure 8 shows the relation with all data for Lines L1, L2, M1, M2, N1, and N2, as shown in Figure 2d,e. For comparison, the results for the endocuticle in the fixed finger’s exoskeleton [33] and for white areas in the claws (large, medium, and small denticles on the movable finger and fingertip) [38] were also plotted. Data lying on a straight line of *H*^3^/*E*^2^, as shown in Figure 8, indicate materials with equivalent abrasion-resistance performances [39]. The abrasion resistance near the largest denticle surface is higher than that in other areas and is almost the same as that in the surface of the large denticle on the fixed finger. This means that the largest denticle on the movable finger and the two large denticles on the fixed finger have the highest abrasion resistance in the claw exoskeleton. The abrasion resistance of the denticle valley area corresponds to that of the small denticle on the fixed finger.

**Figure 9:**
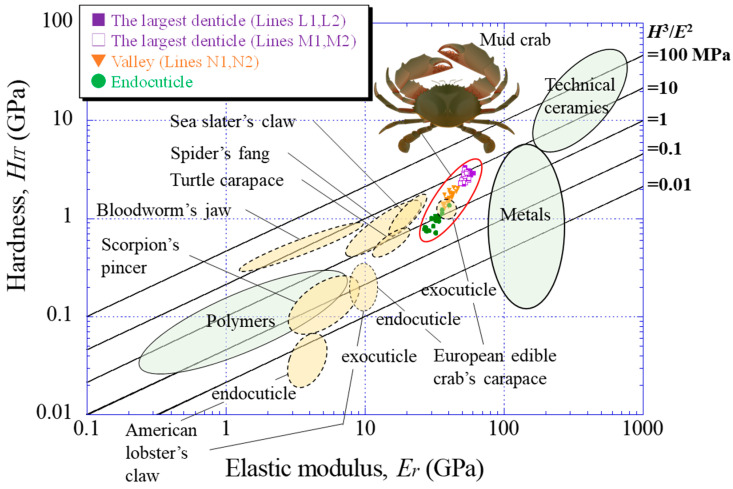
Comparison of the abrasion resistance of the mud crab’s claw with that of engineering materials [39] and various organisms (European edible crab’s carapace [9]; Spider’s fang [11]; Sea slater’s claw [25]; Bloodworm’s jaw [35]; American lobster’s claw [39]; Scorpion’s pincer [40]; Turtle carapace [41]).

Finally, the results are shown on the *H_IT_*–*E_r_* map in Figure 9, including data for engineering material families (technical ceramics, metals, and polymers) and other organisms (American lobster claw [40], European edible crab carapace [9], bloodworm jaw [36], spider fang [11], scorpion pincer [41], turtle carapace [42], and sea slater claw [26]). Here, for Lines L1, L2, M1, M2, N1, and N2, only the results for a range of 500 μm from the surface were plotted. The endocuticle denotes the results for a range of 500 μm from the surface of the fixed finger’s exoskeleton [33]. The *H_IT_*–*E_r_* balance of the largest denticle is higher than the exoskeleton of other organisms. The balance of the endocuticle of the mud crab’s claw is slightly inferior to that of the exocuticle of the European edible crab’s carapace, but its balance is much better than that of the endocuticle of the same crab’s carapace. Furthermore, the data for the largest denticle are much better than those for all polymers and are comparable to soft ceramics and hard metallic alloys. The property map reveals that the values of *H*^3^/*E*^2^ in the largest denticle surface of the mud crab are superior, and the *H_IT_*–*E_r_* balance is in the top class among organisms.

### 4.3. Outlook

The relationship between the microstructure and mechanical properties of living organisms can be investigated using the approach shown in this paper. Since nanoindentation and Vickers tests are methods to examine local mechanical properties, as a next step we need to perform mechanical property tests (tensile test, compression test, fracture test, abrasion test, etc.) on bulk specimens. However, the exoskeleton of crustaceans is thin and curved, making it very difficult to extract specimens. This is a future issue.

The tissue structure of the denticle of the mud crab’s claw may provide ideas for developing novel high-performance materials. In the studies of the denticles of crustaceans [27,29], the microstructures were oriented perpendicular to the surface. For example, in the case of two anomuran crabs, *Paralithodes camtschaticus* and *Paralomis birsteini*, and three brachyuran crabs, *Chionoecetes opilio*, *Callinectes sapidus*, and *Cancer borealis*, the denticle was a twisted pattern structure stacked perpendicular to the surface [29]. The denticle of the coconut crab had a columnar structure perpendicular to the surface [27]. In short, although the tissue patterns are different, the microstructure in the denticle ran perpendicular to the surface. On the other hand, the denticle of the mud crab had a twisted structure stacked parallel to the surface. With this tissue structure, even if a crack occurs inside or on the surface of the denticle, it is difficult for the crack to propagate inside. In the field of materials science and engineering, this is called delamination fracture of a crack-arrester type [43], and it significantly improves the toughness of the material [44,45,46,47]. The denticles of the mud crab had a tissue structure that was never lost even if they cracked during pinching. Design guidelines for improving the toughness of materials through such structures have been adopted for laminate composites [44,45,46] and ultrafine elongated grain structure steels [43,47], but the development of materials with the twisted plywood-pattern structure seen in the denticles has not yet been realized. In the future, as 3D printing technology improves, it may be possible to produce materials with a denticle-like tissue structure. In this case, it will be necessary not only to reproduce the twisted plywood structure, but also to optimize the hardness ratio and volume ratio of the hard (inorganic part) and soft (organic part) materials as a composite. In addition, the crab is designed to optimize not only the tissue structure of the denticle and exoskeleton, but also the function of the entire claw. In the future, we will proceed with a stress analysis study using a numerical simulation (finite element analysis) of the mud crab pinching predators and prey. Finally, we would like to develop bio-inspired materials by understanding the role of each denticle, the tissue structure, the mechanical properties, and the morphology of the claws and by using additive manufacturing.

## 5. Conclusions

The striation patterns originating from a twisted plywood structure parallel to the surface were observed throughout the largest denticle on the movable finger of the mud crab’s claw. However, a significant difference in mechanical properties between the denticle and valley areas without phosphorus existed. This was due to the change in microstructure from dense to coarse. The largest denticle had local mechanical properties of (*H_IT_*, *E_r_*) = (2.5 GPa, 50 GPa), and the area adjacent to the cells was lower. The valley area was (*H_IT_*, *E_r_*) = (1.5 GPa, 38 GPa), and the area adjacent to the cells became lower. These areas adjacent to the cells were the endocuticle layer with phosphorus. This layer was visible as an area with different levels of contrast via SEM observation, and the endocuticle thickness of the denticle valley (250 μm) was five times thicker than that of the denticle peak (50 μm). The abrasion resistance (*H_IT_*, *E_r_*) = (3.0–3.4 GPa, 55–60 GPa) near the largest denticle surface was higher than that in other areas and was almost the same as that in the surfaces of the large denticle on the fixed finger. The abrasion resistance of the denticle surface was in the top class among organisms.

## Figures and Tables

**Table 1:** EDS area scan results of the inner and innermost areas of Lines L1 and N1. Here, the results show the average weight %. Referred data (Fingertip [33]; Large denticle on the fixed finger [38]; Claw outer side [33]).

Area	Site	Ca	Mg	P	C	O	Na	Cl	S
Line L1 (peak)	Inner (denticle) Innermost (endocuticle)	29.1 23.7	1.4 1.4	0.0 1.0	15.8 24.7	52.6 48.4	0.9 0.6	0.0 0.0	0.1 0.2
Line N1 (valley)	Inner (denticle) Innermost (endocuticle)	26.7 24.5	1.9 2.2	0.0 0.8	18.4 20.7	52.1 50.9	0.8 0.8	0.0 0.0	0.1 0.1
Fingertip	Surface Innermost (endocuticle)	30.3 24.9	0.9 2.2	0.0 0.7	16.2 20.9	51.7 50.4	0.9 0.8	0.0 0.1	0.0 0.1
Large denticle on fixed finger	Inner (denticle) Innermost (endocuticle)	29.8 25.5	1.4 2.3	0.0 0.6	15.6 22.1	52.1 48.6	1.0 0.7	0.1 0.2	0.0 0.1
Claw outer side	Endocuticle Endocuticle	24.8 23.5	2.0 1.9	0.8 0.7	20.8 21.4	50.6 51.3	0.8 0.8	0.1 0.2	0.1 0.2

## Data Availability

Data are contained within the article.
